# Algal Turf Sediments and Sediment Production by Parrotfishes across the Continental Shelf of the Northern Great Barrier Reef

**DOI:** 10.1371/journal.pone.0170854

**Published:** 2017-01-25

**Authors:** Sterling B. Tebbett, Christopher H. R. Goatley, David R. Bellwood

**Affiliations:** College of Science and Engineering and ARC Centre of Excellence for Coral Reef Studies, James Cook University, Townsville, Queensland, Australia; Leibniz Center for Tropical Marine Ecology, GERMANY

## Abstract

Sediments are found in the epilithic algal matrix (EAM) of all coral reefs and play important roles in ecological processes. Although we have some understanding of patterns of EAM sediments across individual reefs, our knowledge of patterns across broader spatial scales is limited. We used an underwater vacuum sampler to quantify patterns in two of the most ecologically relevant factors of EAM sediments across the Great Barrier Reef: total load and grain size distribution. We compare these patterns with rates of sediment production and reworking by parrotfishes to gain insights into the potential contribution of parrotfishes to EAM sediments. Inner-shelf reef EAMs had the highest sediment loads with a mean of 864.1 g m^-2^, compared to 126.8 g m^-2^ and 287.4 g m^-2^ on mid- and outer-shelf reefs, respectively. High sediment loads were expected on inner-shelf reefs due to their proximity to the mainland, however, terrigenous siliceous sediments only accounted for 13–24% of total mass. On inner-shelf reef crests parrotfishes would take three months to produce the equivalent mass of sediment found in the EAM. On the outer-shelf it would take just three days, suggesting that inner-shelf EAMs are characterised by low rates of sediment turnover. By contrast, on-reef sediment production by parrotfishes is high on outer-shelf crests. However, exposure to oceanic swells means that much of this production is likely to be lost. Hydrodynamic activity also appears to structure sediment patterns at within-reef scales, with coarser sediments (> 250 μm) typifying exposed reef crest EAMs, and finer sediments (< 250 μm) typifying sheltered back-reef EAMs. As both the load and grain size of EAM sediments mediate a number of important ecological processes on coral reefs, the observed sediment gradients are likely to play a key role in the structure and function of the associated coral reef communities.

## Introduction

Sediments can be found on all coral reefs and are derived from a number of sources ranging from on-reef production via bioerosion to inputs from terrestrial systems [1–3]. While sediment is a ubiquitous feature of coral reefs, concern has been raised about the potential ecological effects of increased sediment inputs from terrestrial sources [4–7] and coastal activities such as dredging [8,9]. Although many corals exist in high sediment locations [10,11], sediments are widely regarded as potentially detrimental to coral reefs [9,12–14] and have an array of negative ecological effects on coral reef organisms [8,15–18], especially when in suspension [17–21]. However, suspended sediment are highly variable in terms of concentration, and on mid- and outer-shelf reefs only account for a small proportion of sediment associated with these reefs [3,22–24].

Most sediment in reef systems is in the off-reef apron, i.e. as sand or mud around the reef. However, almost every hard surface on the reef is also covered with some sediment, and it is these sediments, especially those within the epilithic algal matrix (EAM), that may have the most long-lasting effect on coral reef organisms [14,25]. It is these algal turf-based sediments in the EAM that are the focus of this study. The EAM is a widespread benthic feature on coral reefs, comprising short turfing algae, detritus, cryptofauna, microalgae, microbes and sediment [26–28]. Sediment can become trapped among the algal filaments of the EAM for long periods [25] as the complex structure of the algae reduces surface water flow increasing deposition [29,30]. Both the total load of sediment and the specific grain size of sediments trapped in the EAM can affect a variety of coral reef organisms. Sediments can reduce coral settlement [31,32] and the feeding rates of herbivorous and detritivorous fishes [16,33–35]. By affecting vital processes such as coral recruitment and herbivory, the total load and size of inorganic sediments can directly affect benthic communities and consequently the resilience of coral reefs to further anthropogenic disturbances.

Across the continental shelf of the Great Barrier Reef (GBR) there are numerous ecological gradients in terms of the community composition of benthic components, corals, cryptofauna and fishes [36–41]. Water quality and suspended sediment concentration also vary across the continental shelf due to decreases in terrestrial influences with distance from the land, and differences in hydrodynamic activity [12,42,43]. Many ecological gradients are associated with, or driven by, this cross-shelf variation in physical factors [37,39,44]. Some ecological gradients may also be driven by variation in EAM sediment loads [36,37,40,41]. Across smaller spatial scales (reef habitats), distinct patterns of EAM sediments have been documented [45,46], with evidence that these patterns may mediate key ecological processes, such as herbivory [16,46–48]. As a consequence, gradients in EAM sediments may represent an important factor influencing both patterns and processes in cross-shelf benthic communities. It is therefore surprising that we currently lack a quantitative evaluation of EAM sediments across the GBR.

In addition to our understanding of how EAM sediments vary across broad spatial scales, we also have a limited knowledge of the factors which are responsible for maintaining EAM sediment loads on coral reefs. The factors contributing to variation in suspended sediments across broad spatial scales have received some attention [12,43,49], although their links to EAM sediment is unknown. Indeed, recent evidence suggests that suspended sediment and EAM sediment loads may not be correlated [25]. For instance, one factor which may contribute disproportionately to EAM sediments is variation in sediment production and reworking by parrotfishes [50,51]. The importance of this group of reef fishes in sediment dynamics is becoming increasingly apparent [51–53] and, along with other herbivorous fishes, the distribution and abundance of parrotfish species shows marked variation across the continental shelf of the GBR [54].

Parrotfishes can be divided into three distinct functional groups, browsers, scrapers and excavators, each fulfilling a different role on coral reefs [55]. The two functional groups of interest when considering sediment dynamics are scrapers and excavators [56]. Scraping species are predominantly responsible for the reworking of sediment, i.e. ingesting and possibly altering the particle sizes of sediments extracted from the EAM while feeding [50,55]. Excavators produce new sediment through bioerosion and subsequent defecation, as they remove significant amounts of the reef substratum during feeding [50,55]. Cross-shelf variation in the abundance of scraping and excavating parrotfishes, therefore, directly corresponds with changes in the levels of reworking and bioerosion across the GBR [54].

A variety of non-fish coral reef organisms are also associated with the on-reef production of sediment through bioerosion, including sea urchins and endolithic boring organisms such as polychaetes and sponges [1,52,54]. However, on much of the GBR parrotfishes are considered to be the primary bioeroding organisms. On the windward slope of Lizard Island, for example, they account for 83–94% of total bioerosion [1,54,57,58]. It has been suggested that parrotfishes are the primary bioeroders in all shelf habitats across the northern GBR, as bioerosion rates commonly exceed those of endolithic boring organisms and sea urchins in the same locations [54,59]. Furthermore, the sediment produced by parrotfishes is likely to be particularly important in EAM sediment dynamics as a large proportion of the sediment is released directly onto the reef and has the potential to be incorporated into EAMs [60]. By comparing and contrasting sediment production and reworking by parrotfishes with EAM sediment distributions across the same spatial scale we can begin to gain insights into the potential contribution of parrotfishes to EAM sediment dynamics.

While there is some information available pertaining to the dynamics of suspended sediments, and sediment production and reworking across broader spatial scales [12,43,54,61,62], our understanding of the distribution of EAM sediments is currently limited to within-reef scale studies [25,45,47]. Furthermore, patterns of EAM sediment distribution have not previously been compared with major sediment inputs across broad spatial scales. The aim of the present study, therefore, is to quantify two of the most ecologically-relevant factors of EAM sediments (total inorganic load and grain size distributions) across the continental shelf of the northern region of the GBR. In addition, these sediment loads will be compared and contrasted with the rates of sediment production and reworking by parrotfishes to provide an insight into one of the key factors which may shape EAM sediment dynamics.

## Materials and Methods

### Ethics statement

This study was conducted in accordance with all permitting requirements of the Great Barrier Reef Marine Park Authority including authorisation to collect sediments using a vacuum sampler (permit numbers: G12/35057.1 and G13/36627.1). Ethics approval was not required for this study because the sediment collection and processing did not involve vertebrates or cephalopods and data on parrotfishes was sourced from a previously published study. Data are available in the supplementary material (S1 and S2 Tables).

### Sediment collection

**Fig 1:**
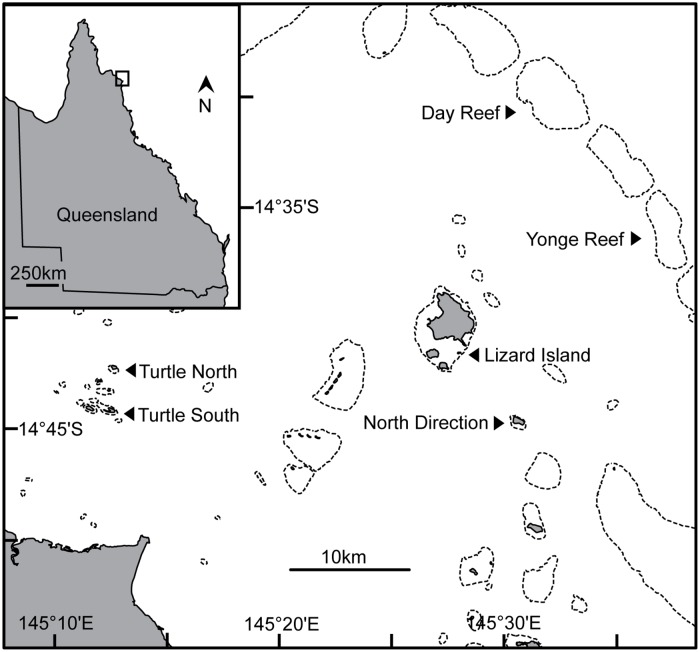
Map of study sites. The northern Great Barrier Reef showing the inner-shelf (Turtle Group), mid-shelf (Lizard Island and North Direction) and outer-shelf (Day and Yonge) reefs sampled.

Sediment samples were collected in the northern region of the GBR (approx. 14′ 40°S) during the summer months between 2012–2015. At each shelf position; inner-, mid- and outer-, two reefs were selected: two islands in the Turtle Group on the inner-shelf, Lizard Island and North Direction Reefs on the mid-shelf, and Day and Yonge Reefs on the outer-shelf (Fig 1). At each reef 10 particulate samples were collected from the exposed reef crest and sheltered back reef, all in 2–5 m of water. On inner-shelf reefs the reef crest is indistinguishable from the reef slope [54], consequently samples were collected from a combined reef crest/slope habitat on inner-shelf reefs. Care was taken to ensure similar suitable EAM-covered surfaces were sampled at each site so that the selected EAMs were representative of open grazed areas. Sampling was conducted by haphazardly placing a 58 cm^2^ PVC ring on a suitable EAM-covered surface and collecting the enclosed particulates using a submersible electronic vacuum sampler [28,63].

Suitable EAM sampling surfaces were flat (< 15° from horizontal) areas of consolidated reef substratum covered by an EAM, following [45,48]. Each surface had to be free of large sediment-retaining pits, macroalgae and encrusting organisms, evenly covered by short algal turfs (< 5 mm in height) and outside the territories of damselfish [14,25,45,47,48]. EAMs of this nature were abundant at all sampling sites across the shelf *cf*. [37]. Sample sites were selected by the same person at all sites to ensure consistency of methods and surface selection. As this sampling methodology was applied at each site, it allowed sediments in flat EAMs, in the same two habitats in each reef to be compared across the three different shelf positions. Samples, therefore, were representative of open grazed EAMs across the GBR.

### Particulate sample treatment

Each sample was settled in a 9 l container before being transferred to a 120 ml sample jar. A minimum of three hours was left before decanting the water from samples to allow particulate material > 10 μm to settle [64]. To remove salts, each sample jar was rinsed with freshwater three times, with a 3 h sediment settling period between each rinse. Samples were then wet sieved through 2 mm stainless steel mesh. All particulate material less than 2 mm was considered sediment (sands, silts and clays; ISO 14688–1:200). To remove organic material, samples were bleached for three days in a 10% sodium hypochlorite solution (NaHClO_4_). The bleaching protocol was repeated three times to ensure all organic material was removed [63]. After bleaching, each sample was rinsed three times with freshwater to remove residue and salts, allowing a standard 3 h settling period between rinses. The bleached sediment was then dried to a constant weight at 60°C and weighed. Samples were dry sieved through a sieve stack (2000 to 63 μm) and the size fractions individually weighed.

To determine the proportion of EAM sediments on inner-shelf reefs derived from terrestrial sources, sediment samples were treated with 5% hydrochloric acid (HCl) to remove carbonates [65], rinsed three times with freshwater, and dried, as above. Samples were then weighed to determine the proportions of carbonates to silicates.

### Statistical analysis

Differences in total EAM sediment loads were examined using a generalised linear mixed effects model (GLMM) with a Gamma distribution and log link function. Shelf position and reef habitat (crest and back) were treated as fixed effects while reef was treated as a random factor nested within shelf position. Models were simplified based on the corrected Akaike Information Criterion (AICc [S3 Table]). Model fit was assessed using residual plots; all of which demonstrated homoscedasticity. Statistical modelling was performed in R [66] using the *lme4* [67] and *AICcmodavg* [68] packages.

Grain size distribution patterns were inspected using a non-metric multidimensional scaling (nMDS) ordination based on a Bray Curtis similarity matrix of standardised and log (*x*+1) transformed data, and differences tested using a permutational multivariate analysis of variance (PERMANOVA). Data were standardised and log (*x*+1) transformed to account for differences in total mass and to reduce the effects of outliers [69]. In the PERMANOVA design, shelf position and reef habitat were treated as fixed factors with an interaction term, while reefs were treated as a random factor nested within shelf position. Following the PERMANOVA, pair-wise tests were performed to determine within-factor differences. For the PERMANOVA the assumption of homogeneity of dispersions was tested using permutational analyses of multivariate dispersions (PERMDISPs). All multivariate analyses were performed using PRIMER 6.0 PERMANOVA+.

### Sediment loads vs. sediment produced and reworked by parrotfishes

To explore the potential contribution of parrotfishes to the observed patterns of EAM sediments, the quantities of sediment reworked or produced through bioerosion by parrotfishes were compared to EAM sediment loads across the continental shelf (S1 Text). Standard units (kg m^-2^) are used for clarity where m^2^ refers to the total census areas not the proportion of the reef covered in EAM. Rates of sediment production and reworking by parrotfishes (in kg m^-2^ year^-1^) were sourced from a study conducted in the GBR which quantified the distribution and functional roles of 24 parrotfish species [54]. The parrotfishes were surveyed in 1998–1999 [54] using timed swims to minimise diver effects [70]. The abundance and community composition of parrotfishes on the GBR has remained largely unchanged since this time [71]. Parrotfish surveys and sediment collection were both performed prior to the major disturbances to the northern GBR (cyclone and bleaching events in 2015–2016). The parrotfish study [54] was from the same region (northern GBR) along the same transect (inner-, mid- and outer-shelf reefs around Lizard Island), and from sites that were the same or close to those used for sediment sampling.

## Results

**Fig 2:**
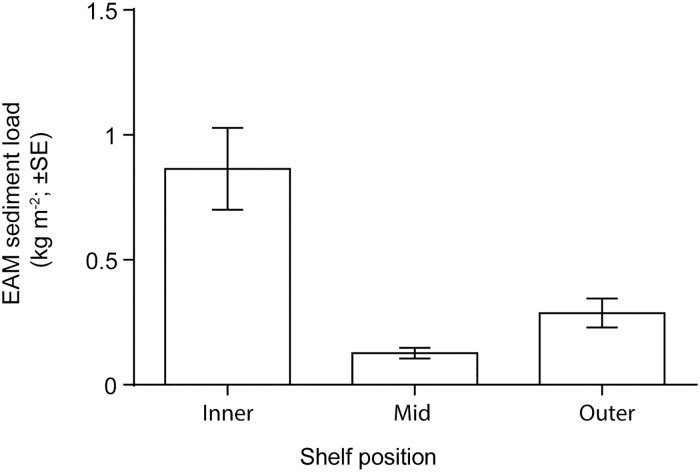
Sediment loads in the epilithic algal matrix across the northern Great Barrier Reef. Samples were collected from back reef and reef crest habitats from inner- (n = 40), mid- (n = 36) and outer-shelf (n = 39) reefs.

Clear differences in EAM sediment loads were recorded among shelf positions (Fig 2). The average sediment load on inner-shelf reefs (864.1 ± 163.8 g m^-2^; mean ± SE) was nearly seven times higher than on mid-shelf reefs (126.8 ± 21.7 g m^-2^), and three times higher than on outer-shelf reefs (287.4 ± 57.8 g m^-2^; Fig 2). Inner-shelf loads were significantly higher than both mid-shelf reefs (GLMM; *p* < 0.001) and outer-shelf reefs (GLMM; *p* < 0.001) (S4 Table). The model containing only shelf position had the lowest AICc (S3 Table), suggesting that neither the shelf position × reef habitat interaction nor habitat had a substantial effect on EAM sediment loads. On the inner-shelf reef crest EAM sediments were composed, on average (± SE), of 13.13 ± 1.42% silicates, while on inner-shelf back reefs EAM sediments contained an average of 23.79 ± 3.24% silicates.

**Fig 3:**
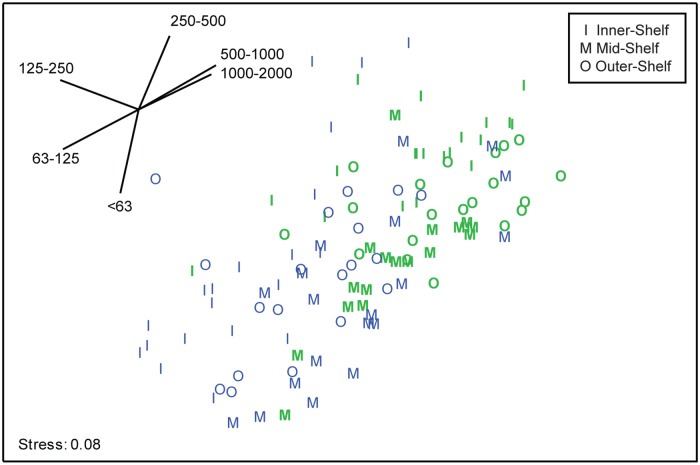
Nonmetric multidimensional scaling plot of grain size distributions in EAM sediment samples. The samples are from back reefs (blue) and reef crests (bold green) across the continental shelf of the northern Great Barrier Reef; vectors represent grain sizes in μm and indicate the source of any differences among samples.

In contrast to mean EAM sediment loads, which differed among shelf positions but not between habitats, the particle size distributions of these sediments differed between habitats within a reef and between reefs, but not among shelf positions (Fig 3; S5 Table). The PERMANOVA analysis found significant differences in grain size distributions between reef habitats (PERMANOVA: Pseudo-*F*_1,114_ = 25.174, *p*[perm] < 0.05), and between individual reefs within the same shelf position (PERMANOVA: Pseudo-*F*_3,114_ = 4.811, *p*[perm] < 0.001). Pairwise analyses revealed that the significant difference in grain size distribution between reefs was a result of differences between individual outer- (*t* = 3.145, *p*[perm] < 0.001) and mid-shelf reefs (*t* = 2.321, *p*[perm] < 0.05) only. The PERMDISP analysis found that grain size distributions from back reef habitats were more dispersed than distributions from reef crest habitats (PERMDISP: *F*_2,113_ = 4.146, *p*[perm] < 0.05). However, habitat separation as revealed by the PERMANOVA was clear based on a greater abundance of coarse sediments (> 250 μm) on reef crests, while back-reefs had more fine sediments (< 250 μm) (Fig 3; S6 Table).

**Fig 4:**
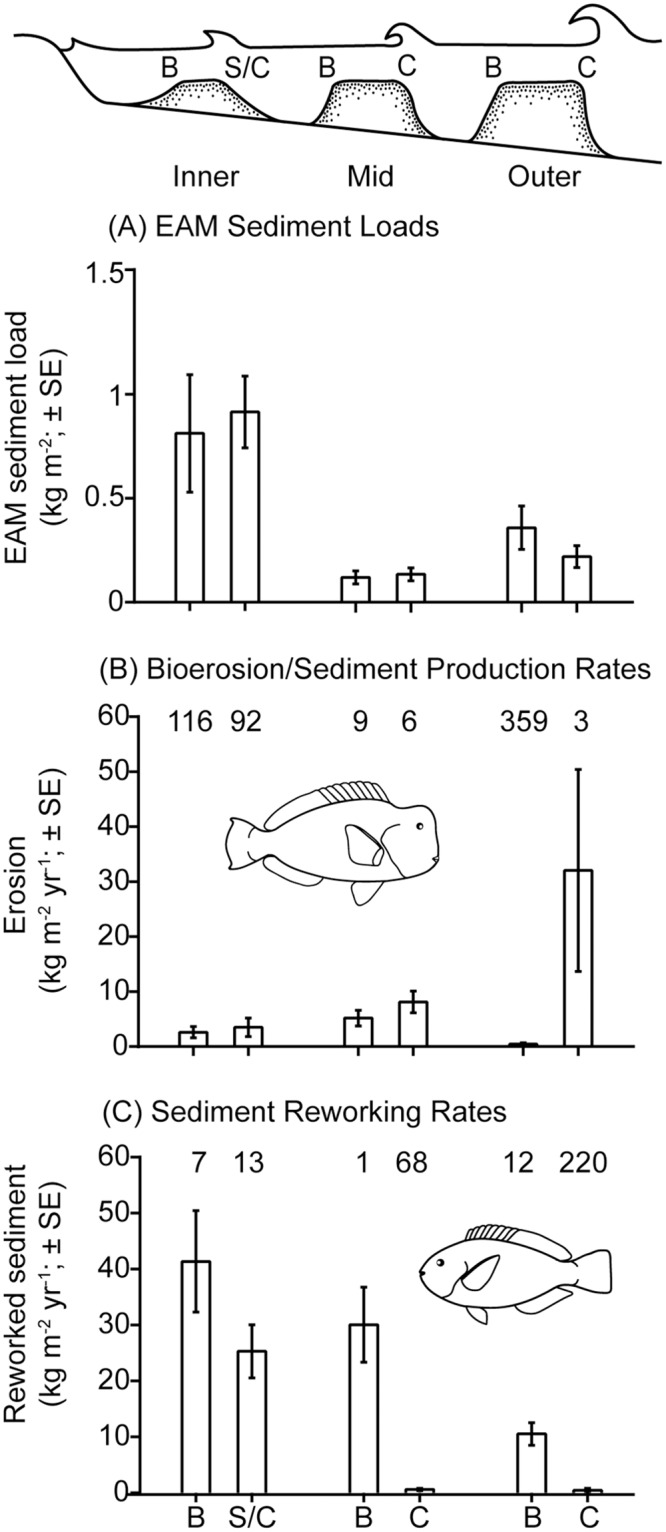
EAM sediment loads versus sediment production and reworking by parrotfishes. Levels of (A) sediment in the EAM, (B) sediment produced through bioerosion by parrotfishes and (C) sediment reworking by parrotfishes for back reefs and reef crests across the continental shelf of the northern Great Barrier Reef. The numbers above the bars on plots (B) and (C) are the number of days it would take parrotfishes to produce (B) and rework (C) the equivalent amount of sediment to EAM sediment load at each location. B = back reef, C = crest and S/C = combined slope/crest on inner-shelf reefs where the habitats are indistinguishable. Data on parrotfish bioerosion and reworking sourced from [54].

On outer-shelf reef crests parrotfishes produce 88 g m^-2^ day^-1^ of sediment. Given that EAMs in this location contain 220 g m^-2^, parrotfishes could produce the equivalent of all EAM sediments in just three days (Fig 4; Table A in S1 Text). On mid-shelf reef crests it would take parrotfishes six days to produce the equivalent mass of sediment found in EAMs, while on inner-shelf reef crests it would take three months (10 g m^-2^ day^-1^ sediment production vs. 915 g m^-2^ in EAMs [Table A in S1 Text]). On back reefs parrotfish may be especially important in sediment reworking. This is particularly clear on mid-shelf reefs where daily reworking rates (82 g m^-2^ day^-1^) approximate total EAM sediment loads (120 g m^-2^) (Table A in S1 Text).

## Discussion

We found that EAM sediment loads exhibit distinct patterns across the GBR continental shelf. Inner-shelf reefs have markedly higher sediment loads than mid- and outer-shelf reefs. However, no cross-shelf gradients in grain size distributions were apparent. Instead, grain size distributions differed at a smaller scale, i.e. between habitats and individual reefs within the same shelf position. As EAM sediments mediate numerous ecological processes on coral reefs [16,31,72], especially those which involve interactions with the benthos, understanding the variability of sediments across broad spatial scales is particularly important. The sediments, in turn, may also be mediated by ecological processes. In this respect, we highlight how parrotfishes are likely to play a key role in EAM sediment dynamics, especially on outer-shelf reef crests.

The high sediment loads in EAMs on the inner-shelf of the GBR are particularly striking when compared to the loads found on mid- and outer-shelf reefs. Inner-shelf coral reefs differ from mid- and outer-shelf reefs with distinct fish and benthic communities [36,37,39,41]. The sediment loads reflect these differences. Sediments within the EAM directly affect the functioning of coral reefs by mediating processes such as feeding rates of herbivorous/detritivorous fishes [14,16,35] and coral settlement [31,73]. By affecting organisms which characterise coral reefs, the high sediment loads contained within inner-shelf reef EAMs have the potential to play a key role in structuring the coral reef communities that typify the inner-shelf. Essentially, high EAM sediment loads may determine what organisms can settle and persist on inner-shelf reefs, limiting coral reef communities to those organisms which can cope with high sediment loads. For herbivorous fishes, this is marked by assemblages that are dominated by the sediment-tolerant parrotfish, *Scarus rivulatus* [16,34,74].

While high sediment loads affect ecological processes, these ecological processes may, in turn, shape the sediment loads themselves, however, physical processes are also important. The physical processes which characterise inner-shelf coral reefs are again markedly different to mid- and outer-shelf reefs [12,43,49,75]. The high EAM sediment loads on the inner-shelf may be due to: a) differences in the source and rates of delivery of sediments, and b) the propensity of EAMs on inner-shelf reefs to retain sediments. It would be expected that due to the proximity of inner-shelf reefs to the coast, terrestrially-derived, siliceous sediments would be a major constituent of EAM sediment loads. Interestingly, this was not the case. Siliceous sediments only accounted for 13–24%, of the total inorganic EAM sediment load on inner-shelf reefs. This composition is comparable to previous observations of EAMs at Orpheus Island on the inner-shelf of the Central GBR which contained 15–46% siliceous sediment [25]. The composition of EAM sediments contrasts markedly with that of suspended sediments which are composed predominantly of fine inorganic siliceous sediments and organic material which flocculate together [12,76]. It is this fine inorganic and organic material which is predominantly resuspended and/or transported in flood plumes and delivered to inner-shelf reefs [12,43,76]. However, while high loads of fine suspended sediments are characteristic of inner-shelf coral reefs it appears that the input of fine siliceous sediments to EAM sediment loads is relatively minor. It appears that there may be a disconnect between sediments suspended in the water column and sediments contained in the EAM [25]. High loads of suspended siliceous sediments may contribute to the fine components of reef EAMs, but they do not drive the high loads of EAM sediments. Clearly, the on-reef production of carbonate sediments was most likely to be the primary source of EAM sediment loads on the inner-shelf reefs examined herein.

Carbonate sediments on reefs are generally coarser than siliceous sediments [25] and are therefore likely to be produced directly on the reef as only fine sediments can be transported over broad distances [12,43,49]. However, rates of on-reef sediment production are limited on inner-shelf coral reefs. Parrotfishes are the chief bioeroding organism on the GBR and on inner-shelf reef crests they only produce about 10 g m^-2^ day^-1^ of sediment. It would take parrotfishes approximately three months to produce the 915 g m^-2^ of sediment found in inner-shelf reef crest EAMs. On the outer-shelf it would take three days (88 g m^-2^ day^-1^ of sediment produced vs. 220 g m^-2^ in the EAM). The contribution of other bioeroding organisms, including endolithic boring organisms and sea urchins, may be higher on inner-shelf reefs than offshore, but they remain a fraction of parrotfish sediment production rates [54,59,62]. However, it should be noted that the skeletal remains of articulated coralline algae and foraminifers [77] as well as bioerosion from fungi, cyanobacteria and sponges [78,79] also contribute significantly to sediment production on coral reefs and may be important in maintaining the patterns observed in our study. In addition, carbonate sediments can be produced directly through the physical breakdown of skeletal remains of organisms but this is likely to occur at a lower rate than bioerosion on the reef [3,54]. Thus, although carbonate sediments are a major component of EAM sediments on the inner-shelf, the inputs of carbonate sediments onto inner-shelf coral reefs appears to be much lower than on mid- and outer-shelf reefs. Lower sediment inputs, yet higher total sediment loads tend to suggest that inner-shelf reef EAMs have a greater propensity to retain sediments once they are trapped among the algal filaments. EAM sediments on inner-shelf reefs may be far less dynamic than those in EAMs on mid-and outer-shelf reefs.

Inner-shelf reefs are not exposed to the same degree of wave energy compared to outer-shelf, and to a lesser extent, mid-shelf reefs [80,81]. On inner-shelf reefs hydrodynamic activity readily resuspends and transports fine, loose sediments from the reef apron [43] and when deposition initially occurs on hard substrates, loose sediments are likely to be resuspended and lost relatively easily. However, once these sediments have bound within algal turfs [82] it is likely that even higher energy levels from hydrodynamic activity would be required to remove them. Indeed, recent evidence from Orpheus Island has demonstrated that EAM sediment loads were temporally stable over a 6 month period [25]. Furthermore, during this period the reef was subjected to a category three cyclone, however, no significant change in EAM sediment loads was detected [25]. Compared to the concentration of suspended sediments on the inner-shelf, which may be highly variable over short time periods [83], EAM sediments are stable and as such could represent a chronic long-term stressor which builds up slowly over time [25]. Unfortunately, the long-term nature of this build up means that increases may go unnoticed until they begin to affect ecological processes [14].

Compared to inner-shelf EAMs, those found on outer-shelf reef crests appear to be fundamentally different due to the high-turnover nature of this environment. On outer-shelf reef crests the on-reef production of sediment is particularly high: in this location bioerosion by parrotfishes approximates total calcification [84]. The parrotfish communities on outer-shelf reef crests produce approximately 88 g m^-2^ day^-1^ of sediment compared to 220 g m^-2^ in the EAM. The high bioerosion rates and subsequent sediment inputs are chiefly driven by the bumphead parrotfish, *B*. *muricatum*, which often defecate in feeding areas (DRB, CHRG pers obs). Much of the sediment produced by *B*. *muricatum* is therefore available for incorporation into the EAM. However, although sediment inputs are high, the retention of sediments within the EAM is likely to be quite low. The outer-shelf of the GBR is very exposed to oceanic swells and associated hydrodynamic activity [80,81] and consequently much of the sediment produced on the reef is likely to be transported away before it can be incorporated into EAMs. A similar conclusion was reached by [80] when examining the distribution of particulate detrital material on mid- and outer-shelf reefs on the GBR. The intermediate sediment loads characteristic of outer-shelf reef crest EAMs are therefore likely to be a result of high levels of sediment input but also high levels of export through hydrodynamics.

The lowest EAM sediment loads were found on mid-shelf coral reefs. Similarly low EAM sediment loads have previously been reported for mid-shelf coral reefs on the GBR [45,47]. The low EAM sediment loads on mid-shelf reefs could be a result of lower sediment inputs, and/or higher rates of export from the EAM due to differences in reef geomorphology or ecological processes. Mid-shelf reefs have limited sediment inputs as they lie beyond the shallow inshore sediment-rich resuspension zone, are less closely linked to terrestrial systems, and have lower rates of on-reef sediment production compared to outer-shelf reefs [49,54,85]. Additionally, ecological processes may also contribute to the low EAM sediment loads. Only moderate rates of sediment production via parrotfishes occur on mid-shelf reefs largely due to a lower abundance of *B*. *muricatum* [54]. As mid-shelf reefs are dominated by *C*. *microrhinos*, export of sediment off the reef may also be significant. It is estimated that approximately one third of defecation events by this species take place away from EAM-dominated feeding areas [60]. Additionally, the surgeonfish, *Ctenochaetus striatus*, is likely to contribute significantly to the export of sediment from EAMs to deeper water on mid-shelf reefs [86]. *C*. *striatus* ingests substantial quantities of sediment when feeding on EAMs [34,86] and approximately 80% of defecations occur away from the upper reef crest [86]. *C*. *striatus*-mediated sediment export is largely absent from inner-shelf reefs as this species is only found in high abundances on mid- and outer-shelf reefs where it is one of the most abundant herbivorous/detritivorous coral reef fishes [39,87,88].

Along with hydrodynamic and biological processes the geomorphology of the reefs in question can also shape sedimentary patterns. The wave energy that leaks past the reef crest is critical in sediment transport, but the dissipation and transformation of wave energy is dependent on the tidal elevation and reef morphology [89]. Inner-, mid- and outer-shelf reefs of the GBR have been exposed to different growing conditions during a 100 000 year period of exposure and the Holocene drowning event [75]. The present characteristics of the reefs were acquired during these periods [75]. GBR reefs can be in different growing stages and once a reef reaches sea-level it transitions from reef growth to reef senility [3,75]. Indeed, the growth of most inner-shelf reefs has slowed since the mid-Holocene with many reefs in a stage of senility rather than growth [90]. As reefs grow the hydrodynamic and sedimentary regimes change, resulting in differences in key reef processes [3]. As a consequence the morphology of individual reefs may have influenced the observed sediment patterns documented herein.

Furthermore, it must be noted that the current study only represents a single snapshot in time of the documented cross-shelf EAM sediment patterns. Sampling over temporal time spans would be necessary to see if changes were occurring; the results of the current study, however, provide a baseline for future work. In addition, comparing static values (EAM sediment loads) to rates of sediment production (parrotfish bioerosion) can only highlight the potential importance of this particular sediment input at different locations. It is currently unclear how suspended sediment loads and parrotfish sediment production actually relate to the amount of sediment which is retained within the EAM. EAM sediment loads have the potential to represent a chronic long-term stressor on coral reefs [14,25] and consequently further work towards understanding how other sedimentary processes relate to the loads of EAM sediments would be valuable.

In addition to total EAM sediment loads, the particle size of the EAM sediments can directly affect ecological processes including herbivory/detritivory [16,34], coral settlement [32,91] and algal turf development [72,92]. However, while EAM sediment loads exhibited distinct cross-shelf patterns, this was not the case for the grain size distribution of sediments. Instead, grain size distributions differed predominantly between reef habitats. The difference in grain size distributions between habitats is likely to be driven predominantly by differences in hydrodynamics and reef geomorphology, at within-reef scales. Coral reef crests are exposed to far greater hydrodynamic activity compared to sheltered back-reefs [48,93]. Due to the variation in water movement, as a result of reef morphology, finer sediment is likely to be transported from exposed locations to sheltered back-reefs, where it can be deposited as water movement is reduced [3]. Such a link has been described between fine particulate detrital material and hydrodynamic activity [80].

Hydrodynamic forces and differences in reef morphology may also explain the differences between reefs in the same shelf position. Hydrodynamic energy decreases towards the coast [94], and although inner-shelf reefs are in the resuspension zone, they experience more consistent hydrodynamic forces, which may reduce differences in grain size distributions among individual reefs. Mid- and outer-shelf reefs are likely to be exposed to much more variable hydrodynamic environments [80,81] and this hydrodynamic variability can explain some of the variation in grain size distributions between individual reefs in the same shelf position. As reef morphology affects hydrodynamics at a reefal scale [3,89], if the inner-shelf reefs examined were morphologically similar this may also explain why no differences in sediment distributions were detected between them. However, regardless of shelf position, it appears that high energy reef crest EAMs accumulate coarse sediments (by losing fine sediments) while low energy back-reef EAMs accumulate finer sediments.

The reworking of sediment by parrotfishes also has the potential to contribute to the general patterns documented for EAM grain size distributions. When parrotfishes rework sediments they grind them in their pharyngeal jaws, potentially reducing the size of particles [50], a process which occurs at greater rates on back reefs compared to reef crests at all locations across the continental shelf [54]. Parrotfishes are therefore likely to contribute to the accumulation of finer sediments in back-reef habitats. In addition, parrotfishes produce sediment at greater rates on reef crests than on back-reefs at all locations across the continental shelf, and these sediments are largely composed of coarser grain sizes [54]. However, as parrotfish release sediment in the water column they indirectly assist in the export of fine material as hydrodynamics may transport these finer sediments over 100s of meters away from the point of release, most likely to more sheltered reef habitats [50]. A larger proportion of the courser sediment is likely to settle directly onto the benthos where it can be incorporated into the EAM.

On some reefs parrotfishes are likely to play a substantial role in EAM sediment dynamics, particularly in terms of sediment production on the outer-shelf. However, hydrodynamic activity, reef geomorphology and other physical processes are likely to be pivotal in structuring EAM sediment patterns. Unfortunately we do not know if the broad scale patterns of EAM sediments documented herein are temporally stable or how increased sediment inputs [4,95,96] may relate to the long term accumulation of EAM sediments. However, as EAM sediments play a major role in a number of ecological processes on coral reefs, the observed patterns are likely to be important in structuring ecological gradients across multiple spatial scales.

## Supporting Information

S1 Table Raw data: inorganic EAM sediment loads across the northern Great Barrier Reef. (PDF)

S2 Table Raw Data: percentages of EAM sediment loads in each grain size fraction (μm). (PDF)

S3 Table Comparison of GLMMs used to examine differences in EAM sediment loads. Models are compared using the corrected Akaike Information Criterion (AICc). Shown are degrees of freedom (df), model maximum log-likelihood (logLik), AICc, change in AICc (Δ) and AICc weight (wAICc).
(PDF)

S4 Table Summary of GLMM results used to examine differences in EAM sediment loads. The generalised linear mixed effects model was based on a GAMMA distribution with a log link and contained shelf position as a fixed effect and individual reef as a random effect. SE = standard error, df = degrees of freedom.
(PDF)

S5 Table Summary of PERMANOVA results. The PERMANOVA was based on a Bray-Curtis similarity matrix of standardised, log (χ + 1)-transformed sediment grain size data.
(PDF)

S6 Table Grain size distributions of EAM sediments. (PDF)

S1 Text Parrotfish sediment production/reworking calculations and results. (PDF)
