# Supplementary material for: Algal Turf Sediments and Sediment Production by Parrotfishes across the Continental Shelf of the Northern Great Barrier Reef
Source: PLoS One. 2017 Jan 25;12(1):e0170854. doi: 10.1371/journal.pone.0170854 (PMC5266265; doi:10.1371/journal.pone.0170854)
Supplement: S1 Table — (PDF) [file pone.0170854.s001.pdf]

S1 Table. Raw data: inorganic EAM sediment loads across the northern Great Barrier Reef.

Shelf Position	Habitat	Reef	Sediment Load (g m^{-2})
Inner	Back	Turtle North	268.635
Inner	Back	Turtle North	231.159
Inner	Back	Turtle North	167.137
Inner	Back	Turtle North	257.841
Inner	Back	Turtle North	5546.032
Inner	Back	Turtle North	146.015
Inner	Back	Turtle North	922.190
Inner	Back	Turtle North	64.521
Inner	Back	Turtle North	997.797
Inner	Back	Turtle North	131.761
Inner	Crest	Turtle North	2513.376
Inner	Crest	Turtle North	2953.004
Inner	Crest	Turtle North	499.862
Inner	Crest	Turtle North	455.793
Inner	Crest	Turtle North	248.924
Inner	Crest	Turtle North	1257.721
Inner	Crest	Turtle North	230.918
Inner	Crest	Turtle North	587.244
Inner	Crest	Turtle North	999.690
Inner	Crest	Turtle North	96.781
Inner	Back	Turtle South	217.542
Inner	Back	Turtle South	196.488
Inner	Back	Turtle South	618.075
Inner	Back	Turtle South	200.293
Inner	Back	Turtle South	446.015
Inner	Back	Turtle South	224.755
Inner	Back	Turtle South	451.627
Inner	Back	Turtle South	2475.693
Inner	Back	Turtle South	1143.863
Inner	Back	Turtle South	1553.503
Inner	Crest	Turtle South	1301.412
Inner	Crest	Turtle South	483.285
Inner	Crest	Turtle South	777.587
Inner	Crest	Turtle South	594.750
Inner	Crest	Turtle South	1351.179
Inner	Crest	Turtle South	1327.871
Inner	Crest	Turtle South	835.652
Inner	Crest	Turtle South	95.111
Inner	Crest	Turtle South	178.137
Inner	Crest	Turtle South	1515.786

Mid	Back	Lizard Island	285.332
Mid	Back	Lizard Island	92.556
Mid	Back	Lizard Island	55.037
Mid	Back	Lizard Island	349.904
Mid	Back	Lizard Island	37.493
Mid	Back	Lizard Island	231.414
Mid	Back	Lizard Island	94.762
Mid	Back	Lizard Island	69.651
Mid	Back	Lizard Island	25.657
Mid	Back	Lizard Island	463.948
Mid	Crest	Lizard Island	101.559
Mid	Crest	Lizard Island	446.847
Mid	Crest	Lizard Island	92.546
Mid	Crest	Lizard Island	176.412
Mid	Crest	Lizard Island	149.123
Mid	Crest	Lizard Island	210.840
Mid	Crest	Lizard Island	427.582
Mid	Crest	Lizard Island	161.073
Mid	Crest	Lizard Island	33.433
Mid	Crest	Lizard Island	46.319
Mid	Back	North Direction	31.029
Mid	Back	North Direction	58.616
Mid	Back	North Direction	66.103
Mid	Back	North Direction	23.809
Mid	Back	North Direction	39.129
Mid	Back	North Direction	13.468
Mid	Back	North Direction	299.355
Mid	Back	North Direction	25.751
Mid	Back	North Direction	10.320
Mid	Crest	North Direction	102.928
Mid	Crest	North Direction	21.170
Mid	Crest	North Direction	11.186
Mid	Crest	North Direction	30.410
Mid	Crest	North Direction	120.924
Mid	Crest	North Direction	31.111
Mid	Crest	North Direction	128.738
Outer	Back	Day	387.379
Outer	Back	Day	80.675
Outer	Back	Day	142.170
Outer	Back	Day	181.559
Outer	Back	Day	1362.056
Outer	Back	Day	39.549
Outer	Back	Day	30.440
Outer	Back	Day	188.557

Outer	Back	Day	1404.141
Outer	Back	Day	110.799
Outer	Crest	Day	134.513
Outer	Crest	Day	229.559
Outer	Crest	Day	957.643
Outer	Crest	Day	178.701
Outer	Crest	Day	89.263
Outer	Crest	Day	527.186
Outer	Crest	Day	261.909
Outer	Crest	Day	645.064
Outer	Crest	Day	91.927
Outer	Crest	Day	216.495
Outer	Back	Yonge	198.428
Outer	Back	Yonge	1214.747
Outer	Back	Yonge	90.249
Outer	Back	Yonge	86.535
Outer	Back	Yonge	89.495
Outer	Back	Yonge	211.649
Outer	Back	Yonge	172.204
Outer	Back	Yonge	669.416
Outer	Back	Yonge	155.950
Outer	Crest	Yonge	77.110
Outer	Crest	Yonge	70.570
Outer	Crest	Yonge	163.240
Outer	Crest	Yonge	52.195
Outer	Crest	Yonge	34.700
Outer	Crest	Yonge	35.564
Outer	Crest	Yonge	232.543
Outer	Crest	Yonge	211.590
Outer	Crest	Yonge	52.492
Outer	Crest	Yonge	129.841
