# Supplementary material for: Algal Turf Sediments and Sediment Production by Parrotfishes across the Continental Shelf of the Northern Great Barrier Reef
Source: PLoS One. 2017 Jan 25;12(1):e0170854. doi: 10.1371/journal.pone.0170854 (PMC5266265; doi:10.1371/journal.pone.0170854)
Supplement: S2 Table — (PDF) [file pone.0170854.s002.pdf]

S2 Table. Raw Data: percentages of EAM sediment loads in each grain size fraction (μm).

Shelf	Habitat	Reef	1000-2000	500-1000	250-500	125-250	63-125	<63
Inner	Back	Turtle North	1.21	0.71	6.51	28.67	35.04	27.85
Inner	Back	Turtle North	1.04	2.28	7.66	24.01	36.64	28.36
Inner	Back	Turtle North	4.65	2.31	8.16	30.24	31.86	22.78
Inner	Back	Turtle North	1.10	1.89	5.49	28.63	42.16	20.73
Inner	Back	Turtle North	9.18	12.22	38.08	31.99	6.57	1.96
Inner	Back	Turtle North	0.86	1.36	5.26	21.54	39.31	31.68
Inner	Back	Turtle North	2.21	2.77	10.64	30.59	32.82	20.98
Inner	Back	Turtle North	3.78	1.15	12.35	20.80	35.27	26.65
Inner	Back	Turtle North	0.92	0.76	5.92	28.43	35.56	28.41
Inner	Back	Turtle North	8.76	10.04	18.91	28.45	22.45	11.39
Inner	Crest	Turtle North	22.57	37.69	21.64	7.60	7.45	3.05
Inner	Crest	Turtle North	16.58	40.61	28.29	9.34	3.12	2.05
Inner	Crest	Turtle North	24.45	15.76	16.80	14.78	14.59	13.62
Inner	Crest	Turtle North	20.00	31.30	22.35	10.23	7.89	8.22
Inner	Crest	Turtle North	4.43	16.61	32.09	21.20	14.69	10.98
Inner	Crest	Turtle North	11.99	23.13	32.02	23.03	6.56	3.27
Inner	Crest	Turtle North	4.15	14.72	24.27	17.49	22.05	17.31
Inner	Crest	Turtle North	12.58	26.86	25.38	18.42	8.95	7.81
Inner	Crest	Turtle North	9.95	26.32	28.50	18.26	8.31	8.66
Inner	Crest	Turtle North	13.26	23.69	21.83	13.74	11.22	16.27
Inner	Crest	Turtle South	13.18	32.61	26.43	13.06	7.22	7.50
Inner	Crest	Turtle South	11.63	28.10	25.25	13.94	15.06	6.01
Inner	Crest	Turtle South	13.63	32.43	24.43	13.32	8.98	7.20
Inner	Crest	Turtle South	13.39	42.40	17.58	9.30	13.19	4.14
Inner	Crest	Turtle South	22.70	37.51	21.07	10.49	4.73	3.49
Inner	Crest	Turtle South	19.04	35.61	22.17	11.54	6.55	5.10
Inner	Crest	Turtle South	20.57	34.91	22.97	11.27	6.83	3.46
Inner	Crest	Turtle South	3.47	3.27	6.93	26.66	43.93	15.74
Inner	Crest	Turtle South	14.15	6.46	10.05	28.28	28.23	12.81
Inner	Crest	Turtle South	6.46	17.36	25.80	40.10	7.76	2.52
Inner	Back	Turtle South	2.61	2.96	24.79	16.33	29.86	23.46
Inner	Back	Turtle South	2.66	3.21	8.16	17.37	37.89	30.70
Inner	Back	Turtle South	5.10	9.61	20.35	19.79	19.51	25.64
Inner	Back	Turtle South	3.52	5.03	9.81	11.41	29.60	40.63
Inner	Back	Turtle South	3.49	5.53	16.43	16.84	31.21	26.49
Inner	Back	Turtle South	1.76	1.59	5.67	11.87	32.97	46.15
Inner	Back	Turtle South	4.31	5.71	26.83	16.58	22.34	24.23
Inner	Back	Turtle South	3.29	11.99	47.16	28.57	6.60	2.38
Inner	Back	Turtle South	3.63	16.44	38.20	24.87	10.82	6.05
Inner	Back	Turtle South	8.29	23.24	35.43	27.27	4.51	1.26

Mid	Back	Lizard Island	23.32	31.19	22.63	6.51	5.43	10.93
Mid	Back	Lizard Island	3.53	8.52	16.20	22.18	19.53	30.03
Mid	Back	Lizard Island	7.33	7.46	11.52	12.47	10.22	50.99
Mid	Back	Lizard Island	7.42	7.97	16.16	28.00	15.62	24.84
Mid	Back	Lizard Island	4.71	7.21	11.57	20.43	15.97	40.11
Mid	Back	Lizard Island	18.68	13.93	10.04	12.65	14.60	30.10
Mid	Back	Lizard Island	17.71	17.05	15.28	18.10	12.34	19.51
Mid	Back	Lizard Island	1.24	1.84	5.15	10.92	22.26	58.59
Mid	Back	Lizard Island	8.65	6.62	9.96	13.67	9.82	51.27
Mid	Back	Lizard Island	23.17	25.94	27.75	11.64	3.61	7.89
Mid	Crest	Lizard Island	8.62	9.50	9.46	14.40	13.08	44.93
Mid	Crest	Lizard Island	5.41	16.75	53.34	9.55	5.05	9.91
Mid	Crest	Lizard Island	6.29	38.94	19.75	8.69	6.19	20.14
Mid	Crest	Lizard Island	5.09	21.27	17.89	10.48	10.89	34.38
Mid	Crest	Lizard Island	11.35	36.68	16.18	6.54	7.46	21.79
Mid	Crest	Lizard Island	12.35	38.14	15.91	6.55	8.25	18.80
Mid	Crest	Lizard Island	3.05	18.56	24.59	11.53	9.57	32.70
Mid	Crest	Lizard Island	13.41	24.85	18.39	9.18	10.61	23.56
Mid	Crest	Lizard Island	17.93	31.23	13.72	9.83	6.62	20.66
Mid	Crest	Lizard Island	11.66	15.93	14.81	10.78	10.38	36.45
Mid	Back	North Direction	2.99	3.10	6.81	9.07	15.46	62.57
Mid	Back	North Direction	1.14	9.92	12.50	22.54	23.62	30.28
Mid	Back	North Direction	4.02	4.67	9.23	8.66	11.76	61.65
Mid	Back	North Direction	6.97	11.48	8.24	9.58	16.76	46.97
Mid	Back	North Direction	27.02	26.61	14.01	5.75	5.84	20.77
Mid	Back	North Direction	0.00	4.22	8.32	15.28	20.62	51.55
Mid	Back	North Direction	10.37	16.93	42.82	13.71	8.40	7.77
Mid	Back	North Direction	0.76	5.84	11.06	10.30	21.84	50.21
Mid	Back	North Direction	0.39	8.41	15.26	14.09	18.00	43.84
Mid	Crest	North Direction	5.71	11.20	14.48	11.91	12.47	44.23
Mid	Crest	North Direction	2.70	2.34	5.04	11.34	16.47	62.11
Mid	Crest	North Direction	9.72	23.04	15.99	8.78	8.78	33.70
Mid	Crest	North Direction	4.58	10.63	10.86	13.80	12.98	47.15
Mid	Crest	North Direction	5.12	9.84	15.73	14.60	11.33	43.38
Mid	Crest	North Direction	2.07	5.35	8.69	16.78	13.25	53.86
Mid	Crest	North Direction	7.36	16.58	16.64	16.61	12.44	30.36
Outer	Back	Day	16.77	16.93	21.72	19.85	10.44	14.28
Outer	Back	Day	12.78	12.96	13.96	15.66	13.18	31.45
Outer	Back	Day	14.09	25.49	21.20	14.73	10.73	13.76
Outer	Back	Day	11.29	12.59	11.48	10.85	14.20	39.59
Outer	Back	Day	11.95	11.80	19.52	27.90	16.02	12.82
Outer	Back	Day	12.70	8.98	11.34	18.02	18.86	30.10
Outer	Back	Day	0.00	7.44	15.13	18.00	19.25	40.19
Outer	Back	Day	11.04	13.85	16.84	20.83	17.38	20.06

Outer	Back	Day	6.50	11.84	18.40	31.30	14.87	17.08
Outer	Back	Day	10.88	4.89	10.34	14.33	18.32	41.24
Outer	Crest	Day	17.71	29.15	19.80	9.70	5.03	18.61
Outer	Crest	Day	16.00	32.97	29.07	9.14	3.73	9.09
Outer	Crest	Day	4.62	15.61	36.87	19.83	7.55	15.51
Outer	Crest	Day	19.48	27.68	17.43	10.40	6.17	18.84
Outer	Crest	Day	3.26	17.27	30.30	15.83	8.93	24.41
Outer	Crest	Day	16.35	38.21	27.74	7.41	3.23	7.06
Outer	Crest	Day	40.51	29.23	13.91	5.83	3.68	6.85
Outer	Crest	Day	19.54	34.08	26.17	9.30	3.90	7.01
Outer	Crest	Day	9.15	19.26	20.67	10.89	8.45	31.58
Outer	Crest	Day	11.74	27.27	32.71	11.92	5.93	10.45
Outer	Back	Yonge	1.75	2.75	14.46	37.54	23.29	20.21
Outer	Back	Yonge	0.48	2.23	20.26	50.38	17.39	9.27
Outer	Back	Yonge	1.64	2.33	5.80	12.36	27.72	50.15
Outer	Back	Yonge	5.31	4.79	9.31	18.94	29.41	32.23
Outer	Back	Yonge	1.14	4.37	6.21	13.81	31.21	43.26
Outer	Back	Yonge	1.02	3.08	5.12	14.59	29.45	46.74
Outer	Back	Yonge	6.77	11.58	17.77	13.04	18.82	32.02
Outer	Back	Yonge	3.04	4.46	11.15	23.84	24.82	32.70
Outer	Back	Yonge	4.29	9.09	14.48	20.61	25.42	26.10
Outer	Crest	Yonge	7.41	18.22	14.82	8.34	6.53	44.68
Outer	Crest	Yonge	6.08	26.90	31.17	12.03	6.20	17.63
Outer	Crest	Yonge	23.59	30.11	20.70	8.43	5.28	11.89
Outer	Crest	Yonge	7.82	27.00	25.23	10.05	7.59	22.32
Outer	Crest	Yonge	5.81	13.70	19.51	15.31	12.97	32.69
Outer	Crest	Yonge	1.53	9.79	28.73	19.21	15.34	25.40
Outer	Crest	Yonge	25.44	29.99	17.76	5.70	3.93	17.18
Outer	Crest	Yonge	34.77	26.34	15.48	7.11	4.25	12.05
Outer	Crest	Yonge	12.40	14.35	15.92	11.23	9.72	36.37
Outer	Crest	Yonge	34.01	22.87	14.27	7.39	5.86	15.60
