# Supplementary material for: Algal Turf Sediments and Sediment Production by Parrotfishes across the Continental Shelf of the Northern Great Barrier Reef
Source: PLoS One. 2017 Jan 25;12(1):e0170854. doi: 10.1371/journal.pone.0170854 (PMC5266265; doi:10.1371/journal.pone.0170854)
Supplement: S3 Table — Models are compared using the corrected Akaike Information Criterion (AICc). Shown are degrees of freedom (df), model maximum log-likelihood (logLik), AICc, change in AICc (Δ) and AICc weight (wAICc). (PDF) [file pone.0170854.s003.pdf]

S3 Table. Comparison of GLMMs used to examine differences in EAM sediment loads. Models are compared using the corrected Akaike Information Criterion (AICc). Shown are degrees of freedom (df), model maximum log-likelihood (logLik), AICc, change in AICc (Δ) and AICc weight (wAICc).

**Response variable**	**Variables**	**df**	**logLik**	**AICc**	**Δ**	**wAICc**
EAM sediment load	Shelf	6	-435.48	883.73	0.00	0.66
	Shelf + Habitat	7	-435.38	885.81	2.08	0.23
	Shelf × Habitat	9	-434.50	888.71	4.98	0.05
	Null	4	-440.58	889.52	5.79	0.04
	Habitat	5	-440.45	891.44	7.71	0.01
