# Supplementary material for: Algal Turf Sediments and Sediment Production by Parrotfishes across the Continental Shelf of the Northern Great Barrier Reef
Source: PLoS One. 2017 Jan 25;12(1):e0170854. doi: 10.1371/journal.pone.0170854 (PMC5266265; doi:10.1371/journal.pone.0170854)
Supplement: S4 Table — The generalised linear mixed effects model was based on a GAMMA distribution with a log link and contained shelf position as a fixed effect and individual reef as a random effect. SE = standard error, df = degrees of freedom. (PDF) [file pone.0170854.s004.pdf]

S4 Table. Summary of GLMM results used to examine differences in EAM sediment loads.

The generalised linear mixed effects model was based on a GAMMA distribution with a log link and contained shelf position as a fixed effect and individual reef as a random effect. SE = standard error, df = degrees of freedom.

Response variable	Model used	Predictor variable	Estimate	SE	*t* value	*p* value
**EAM sediment load**	Gamma (GLMM)	Intercept	6.760	0.337	20.039	< 0.0001
		Mid-shelf	-2.063	0.432	-4.774	< 0.0001
		Outer-shelf	-1.133	0.452	-2.507	< 0.05
