# Supplementary material for: Algal Turf Sediments and Sediment Production by Parrotfishes across the Continental Shelf of the Northern Great Barrier Reef
Source: PLoS One. 2017 Jan 25;12(1):e0170854. doi: 10.1371/journal.pone.0170854 (PMC5266265; doi:10.1371/journal.pone.0170854)
Supplement: S5 Table — The PERMANOVA was based on a Bray-Curtis similarity matrix of standardised, log (χ + 1)-transformed sediment grain size data. (PDF) [file pone.0170854.s005.pdf]

**S5 Table. Summary of PERMANOVA results.** The PERMANOVA was based on a Bray-Curtis similarity matrix of standardised and log ($\chi + 1$)-transformed sediment grain size data.

| **Source** | **df** | **SS** | **MS** | **Pseudo-*F*** | $p_{[perm]}$ | **Unique perms** |
|---|---|---|---|---|---|---|
| Shelf | 2 | 1049.6 | 524.8 | 1.426 | 0.294 | 90 |
| Habitat | 1 | 3698.1 | 3698.1 | 25.174 | < 0.05 | 9930 |
| Reef (Shelf) | 3 | 1105.4 | 368.5 | 4.811 | < 0.001 | 9940 |
| Shelf × Habitat | 2 | 1047.4 | 523.7 | 3.562 | 0.137 | 9963 |
| Habitat × Reef (Shelf) | 3 | 441.3 | 147.1 | 1.921 | 0.093 | 9914 |
| Residual | 103 | 7889.6 | 76.598 | | | |
| Total | 114 | 15383 | | | | |
