# Supplementary material for: Algal Turf Sediments and Sediment Production by Parrotfishes across the Continental Shelf of the Northern Great Barrier Reef
Source: PLoS One. 2017 Jan 25;12(1):e0170854. doi: 10.1371/journal.pone.0170854 (PMC5266265; doi:10.1371/journal.pone.0170854)
Supplement: S6 Table — (PDF) [file pone.0170854.s006.pdf]

S6 Table. Grain size distributions of EAM sediments.

	Mean (% ± SE) of total mass in individual grain size fractions (μm)					
**Shelf/ Habitat**	**1000-2000**	**500-1000**	**250-500**	**125-250**	**63-125**	**<63**
Inner/ Back	3.62 ± 0.57	6.04 ± 1.36	17.60 ± 2.95	23.21 ± 1.45	27.15 ± 2.63	22.39 ± 2.74
Inner/ Crest	13.91 ± 1.41	26.37 ± 2.48	22.79 ± 1.41	16.60 ± 1.79	12.37 ± 2.14	7.96 ± 1.10
Mid/ Back	8.92 ± 2.00	11.52 ± 1.93	14.45 ± 2.01	13.98 ± 1.35	14.30 ± 1.37	36.84 ± 4.16
Mid/ Crest	7.79 ± 1.06	19.46 ± 2.76	17.15 ± 2.52	11.26 ± 0.75	10.34 ± 0.73	34.01 ± 3.39
Outer/ Back	7.02 ± 1.25	9.02 ± 1.38	13.92 ± 1.19	20.87 ± 2.27	20.04 ± 1.48	29.12 ± 2.86
Outer/ Crest	15.86 ± 2.50	24.50 ± 1.72	22.91 ± 1.57	10.75 ± 0.90	6.71 ± 0.71	19.26 ± 2.36
