# Supplementary material for: Algal Turf Sediments and Sediment Production by Parrotfishes across the Continental Shelf of the Northern Great Barrier Reef
Source: PLoS One. 2017 Jan 25;12(1):e0170854. doi: 10.1371/journal.pone.0170854 (PMC5266265; doi:10.1371/journal.pone.0170854)
Supplement: S1 Text — (PDF) [file pone.0170854.s007.pdf]

S1 Text. Parrotfish sediment production/reworking calculations and results.

Data on the amount of sediment produced and reworked by parrotfishes each year in kg m^{-2} $year^{-1}$ was sourced from [1]. The study performed by [1] was from the same region (northern GBR) along the same transect (inner-, mid- and outer-shelf reefs around Lizard Island) i.e. from the same sites or nearby sites. To calculate the number of days that it would take parrotfish to produce or rework the equivalent amount of sediment contained in EAMs in each habitat within each shelf position the amount of sediment produced/reworked by parrotfish each year in kg m^{-2} day^{-1} was divided by the amount of sediment contained within the EAM in each habitat within each shelf position in kg m^{-2} using equation 1. Using this equation produced the results contained within Table A. Standard units (kg m^{-2}) are used for clarity and are based on censused reef area. These simple calculations and results are used to highlight the potential importance of parrotfish to EAM sediment dynamics. Particle size distributions and hydrodynamic sorting of sediments released by parrotfish are considered in the discussion.

Equation one:

$y = a/b$

y = number of days taken by parrotfish to produce or rework the equivalent amount of sediment contained in the EAM.

a = EAM sediment load (kg m^{-2}).

b = amount of sediment produced or reworked by parrotfishes (kg m^{-2} day^{-1}).

Table A. **Values used in equation one and the results from calculations.** Data on parrotfish sediment production and reworking sourced from [1].

Shelf position/ Habitat	Average EAM sediment load (kg m^{-2})	Average sediment produced by parrotfish (kg m^{-2} day^{-1})	Days taken to produce EAM sediment load	Average EAM sediment load reworked by parrotfish (kg m^{-2} day^{-1})	Days taken to rework EAM sediment load
Inner/ back	0.813	0.007	116	0.113	7
Inner/ crest	0.915	0.010	92	0.069	13
Mid/ back	0.120	0.014	9	0.082	1
Mid/ crest	0.135	0.022	6	0.002	68
Outer/ back	0.359	0.001	359	0.029	12
Outer/ crest	0.220	0.088	3	0.001	220

Reference

1. Hoey AS, Bellwood DR. Cross-shelf variation in the role of parrotfishes on the Great Barrier Reef. Coral Reefs. 2008;27: 37–47.
